# Secretory Expression of an Alkaline Alginate Lyase With Heat Recovery Property in *Yarrowia lipolytica*

**DOI:** 10.3389/fmicb.2021.710533

**Published:** 2021-08-09

**Authors:** Lu Liu, Zhipeng Wang, Zhihong Zheng, Ze Li, Xiaofeng Ji, Haihua Cong, Haiying Wang

**Affiliations:** ^1^Key Laboratory of Sustainable Development of Polar Fishery, Ministry of Agriculture and Rural Affairs, Yellow Sea Fisheries Research Institute, Chinese Academy of Fishery Sciences, Qingdao, China; ^2^School of Medicine and Pharmacy, Ocean University of China, Qingdao, China; ^3^School of Marine Science and Engineering, Qingdao Agricultural University, Qingdao, China; ^4^College of Food Science and Engineering, Dalian Ocean University, Dalian, China; ^5^College of Advanced Agricultural Sciences, Linyi Vocational University of Science and Technology, Linyi, China

**Keywords:** alginate lyase, heat recovery, *Yarrowia lipolytica*, NaCl-independent, oligosaccharides

## Abstract

Alginate lyase possesses wide application prospects for the degradation of brown algae and preparation of alginate oligosaccharides, and its degradation products display a variety of biological activities. Although many enzymes of this type have been reported, alginate lyases with unique properties are still relatively rare. In the present work, an alginate lyase abbreviated as Alyw203 has been cloned from *Vibrio* sp. W2 and expressed in food-grade *Yarrowia lipolytica*. The Alyw203 gene consists of an open reading frame (ORF) of 1,566 bp containing 521 amino acids, of which the first 17 amino acids are considered signal peptides, corresponding to secretory features. The peak activity of the current enzyme appears at 45°C with a molecular weight of approximately 57.0 kDa. Interestingly, Alyw203 exhibits unique heat recovery performance, returning above 90% of its initial activity in the subsequent incubation for 20 min at 10°C, which is conducive to the recovery of current enzymes at low-temperature conditions. Meanwhile, the highest activity is obtained under alkaline conditions of pH 10.0, showing outstanding pH stability. Additionally, as an alginate lyase independent of NaCl and resistant to metal ions, Alyw203 is highly active in various ionic environments. Moreover, the hydrolyzates of present enzymes are mainly concentrated in the oligosaccharides of DP1–DP2, displaying perfect product specificity. The alkali suitability, heat recovery performance, and high oligosaccharide yield of Alyw203 make it a potential candidate for industrial production of the monosaccharide and disaccharide.

## Introduction

Alginate was a linear heteropoly glucuronic polymer extracted from brown algae, consisting of two sugar monomers linked through 1,4-glycoside, occupying approximately 22–44% of its parts dry weight (Gacesa, 1992). Owing to the favorable gel properties, alginate was comprehensively used in food processing. For instance, it could be applied as a thickener and stabilizer to enhance the viscosity and stability of ice cream (Turquois and Gloria, 2000). However, in view of the serious defect of low bioavailability, the wide application of alginate with high molecular weight (Mw) was greatly restricted (Qian et al., 2020). Interestingly, alginate could be dissociated into smaller-molecular-weight alginate oligosaccharides by enzymatic hydrolysis, displaying a variety of biological functions, such as anti-oxidation, antibacterial activity, antitumor, and immunomodulatory effects (Powell et al., 2013; Pritchard et al., 2017). Therefore, the development of alginate lyase with excellent performance for application in marine drug research possessed considerable theoretical and practical value.

As one variety of polysaccharide lyase (PL), alginate lyase could degrade alginate and generate trehalose oligosaccharides through cutting the 1,4-glycosidic bond between C4 and C5 (Ertesvag, 2015). In consideration of the enzyme’s substrate specificity, high efficiency, and mild reaction conditions, the application of alginate lyase in industrial production, particularly during the manufacture of alginate oligosaccharides, has raised more and more concerns. Moreover, the amino acid sequence analysis results showed that they pertained to PL families of 5, 6, 7, 14, 15, 17, 18, 31, 32, 34, 36, and 39 on the basis of the carbohydrate-active enzyme (CAZy) database (Helbert et al., 2019; Ji et al., 2019), of which majority of the alginate lyases were polyM lyases. So far, although many alginate lyases have been recorded, there were relatively few enzymes with special functions such as high activity, heat resistance, or cold adaptability (Jagtap et al., 2014; Bonugli-Santos et al., 2015; Chen et al., 2016). Li et al. cloned a new type of alginate lyase, AlyPL6, with high enzymatic activity from *Pedobacter hainanensis* NJ-02 and immobilized it on mesoporous titanium dioxide granules to enhance thermal stability (Qian et al., 2020). Yang et al. (2020) acquired the alginate lyase cAlyM and corresponding mutant 102C300C with thermostable as well as high enzymatic activity, which were further successfully expressed in *Pichia pastoris*. We have previously reported that Alyw201, an alginate lyase from *Vibrio* sp. W2, possessed strong pH stability and cold adaptation and exhibited superior salt tolerance performance (Wang et al., 2020). However, alginate lyases with thermal stability and heat recovery performance are still rare in the meantime. In fact, the screening of alginate lyase with stable property in industrial production had significant application prospects. For example, heat resistance was conducive to saving energy, reducing production cost, and lowering pollution risk. The heat-resistant alginate lyase could continuously degrade the substrate at high temperatures, thereby reducing the viscosity of the substrate, which was of great significance to the production of alginate oligosaccharides.

In the present work, a novel alginate lyase Alyw203 was characterized and successfully expressed in *Yarrowia lipolytica*. The prepared enzyme exhibited excellent thermal stability and unique heat recovery performance at 10°C. This article not only provided new insights for exploring enzymes with special functions but also laid a solid foundation for the application of Alyw203 alginate lyase in alginate oligosaccharide manufacturing.

## Materials and Methods

### Materials and Strains

Sodium alginate and alginate stochastically consisting of M block and G block were acquired from BZ Oligo Co., Ltd., Prof. Chi Zhenming from the Ocean University of China provided the expression vector pINA1312 and uracil mutant *Y. lipolytica* URA- strain. A YNB medium composed of 5.0 g/L (NH_4_)_2_SO_4_, 1.8 g/L yeast nitrogen base containing no amino acids, 25.0 g/L agar, and 10.0 g/L glucose was used for *Y. lipolytica* URA-transformant screening (Madzak, 2015). Recombinant alginate lyase production was performed on GPPB media containing 3.0 g/L K_2_HPO_4_, 2.5 g/L yeast extract, 1.5 g/L (NH_4_)_2_SO_4_, 0.2 g/L MgSO_4_, 30.0 g/L glucose, and 2.0 g/L KH_2_PO_4_ (Wang et al., 2020).

### Sequence Analysis

With the sake of appraising the gene encoding Alyw203, the genomic DNA was annotated and sequenced using Novogene. Annotated information about sugar-active enzymes could be obtained by comparing the gene protein sequence with the CAZy database. The sequencing result showed that there was a gene encoding Alyw203 with an open reading frame (ORF) of 1,556 bp (Cantarel et al., 2009). The SignalP 4.0 server was applied for signal peptide analysis^1^. The domain analysis of Alyw203 alginate lyase was conducted with the CDD^2^. Both theoretical isoelectric point (pI) and Mw of the present enzyme were forecasted online^3^. DNAMAN 6.0 was used for multiple sequence alignment among the alginate lyases of the PL7 family.

### Expression and Purification of Alyw203

The gene of Alyw203 bearing the XPR2 signal peptide at its 5′ end was synthesized in the subsequent codon optimization and further transformed into the URA- strain. After incubation in the GPPB fluid nutrient medium for 84 h at 30°C, we detected a positive transformant of alginate lyase activity, of which the strain A32 displayed the optimal activity. The biomass and the activity of alginate lyase were measured at fixed time intervals during the subsequent fermentation of the A32 strain. The measurements above were carried out three times.

After adjusting the pH of the obtained supernatant to 7.5, the protein was purified using the Ni-NTA Sepharose column (GE Healthcare, Stanford, United States). A Hi-Trap desalting column (Amersham Biosciences) was applied to further purify the alginate lyase. The Mw and purity of Alyw203 were detected *via* SDS-PAGE on 12% (w/v) gel. In order to study substrate preference of the current Alyw203 alginate lyase, polyG, polyM, and alginate were used as substrates to research the enzyme activity. As previously reported, Alyw203 activity assay was determined by applying 0.5% (w/v) alginate solution as a substrate (Qin et al., 2018). Alyw203 activity confirmation is achieved by measuring the UV absorption at 235 nm (obs235). During the whole experiment, the usage amount of enzyme to increase obs235 by 0.1 per min was considered as one unit (U) of enzyme activity (Wang et al., 2019).

### Effects of Temperature and pH on Alyw203

With sake of determining the optimal temperature, the Alyw203 alginate lyase-catalyzed hydrolysis reaction was carried out in 8 mM citric acid–NaOH media (pH 10.0) within a temperature scope of 20–60°C. Furthermore, the residual enzyme activity was determined at 45°C to detect the thermostability of the present alginate lyase after culturing for 8 h at 20 to 60°C. For the purpose of confirming the optimum temperature for restoring activity, the enzyme treated in a water bath (100°C, 5 min) is incubated at a gradient temperature (4–50°C) for 30 min, and the corresponding activity is measured. In addition, the enzyme (preboiled for 5 min) was cultured at 10°C for different periods (5–50 min) to assess the influence of cold treatment time on the recovery of activity. The measurements above were carried out three times.

To confirm the Alyw203’s optimal reaction pH, alginates within the pH scope of 3.0 to 10.0 buffers were applied as substrates. Additionally, following the culturing of current alginate lyase in media within different pHs for 8 h at 45°C, the remaining activity was measured to detect Alyw203’s pH stability. The measurements above were carried out three times.

### Effects of Ions and NaCl on Activity of Alyw203

Purified Alyw203 enzyme was incubated with the relevant ions dissolved in alginate at 45°C for 25 min; then the activity was measured to evaluate the influence of these matters on the enzyme activity. During the experiment, metal ion and SDS and EDTA solutions with a concentration of 1 and 10 mM, respectively, were used. Moreover, catalyzing reactions of Alyw203 were carried out in alginate liquor with concentrations of NaCl from 0 to 3.0 M at 45°C. The control group was the original alginate without extra substance. The measurements above were carried out three times.

### End Product Analysis of Alyw203

For the purpose of affirming the degradation products of Alyw203, 10 ml of 0.5% sodium alginate solution with purified alginate lyase (40 U) was incubated for 40 min at 45°C. During the whole experiment, the ultraviolet absorption at 235 nm was measured every 5 min. When the absorbance was stable, the samples were desalted, and the degradation products were identified using TLC and ESI-MS methods as reported (Wang et al., 2019).

## Results

### Sequence Analysis of Alyw203

Firstly, genome DNA of the present alginate lyase from *Vibrio* sp. W2 strain has been sequenced, and the results reveal the existence of a putative alginate lyase encoding gene, Alyw203, which is composed of an ORF of 1,566 bp containing 521 amino acids. Further bioinformatics analysis shows that the theoretical Mw and pI of current mature Alyw203 are 57.0 kDa and 6.09, respectively. The first 17 amino acids are regarded as signal peptides, which correspond to secretion characteristics. A detailed retrieval of the Conserved Domain Database from NCBI reveals Alyw203 is a novel type of alginate lyase belonging to superfamily 2.

**FIGURE 1:**
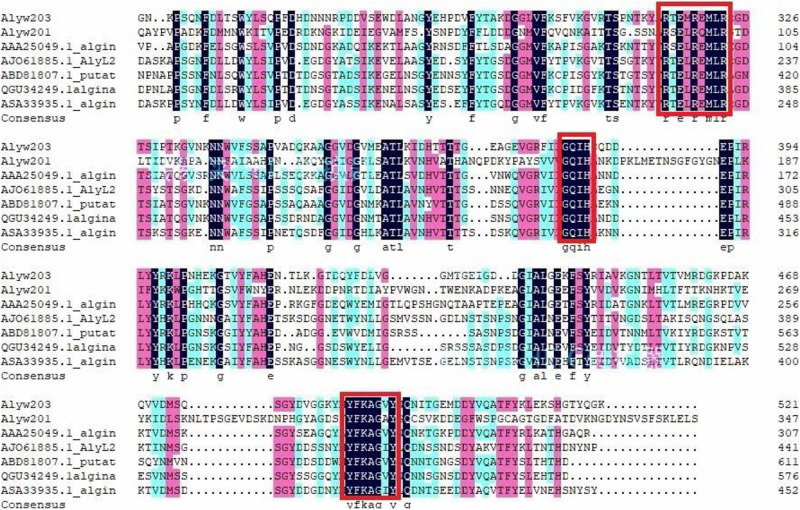
Sequence comparative analysis of Alyw203 with six relevant alginate lyases. The conserved amino acid regions are listed in the red block.

Alyw203 was blasted in NCBI and found to be evolutionarily close with alginate lyases in the PL7 family. According to Alyw203 and other reported sequences of superfamily 7 alginate lyase, a phylogenetic tree has been constructed to further determine the attribution of the current enzyme. Additionally, in the phylogenetic tree, the cluster of deep branches has been constituted among the enzyme from *Klebsiella pneumoniae* (GenBank number: AAA25049.1), the enzyme from *Agarivorans* sp. L11 (GenBank number: AJO61885.1), the enzyme from *Saccharophagus degradans* 2-40 (GenBank number: ABD81807.1) (Kim et al., 2015; Li et al., 2015), the enzyme from *Marinimicrobium* sp. (GenBank number: QGU34249.1), and the enzyme from *Vibrio* sp. (GenBank number: ASA33935.1) (Zhu et al., 2018b; Yan et al., 2019). According to sequence comparison, the Alyw203 includes typical conserved regions of the PL7 family, namely, “QIH,” “RTELREMLR,” and “MYFKAG” (Figure 1).

### Secretory Expression of Alyw203 Alginate Lyase in *Y. lipolytica*

**FIGURE 2:**
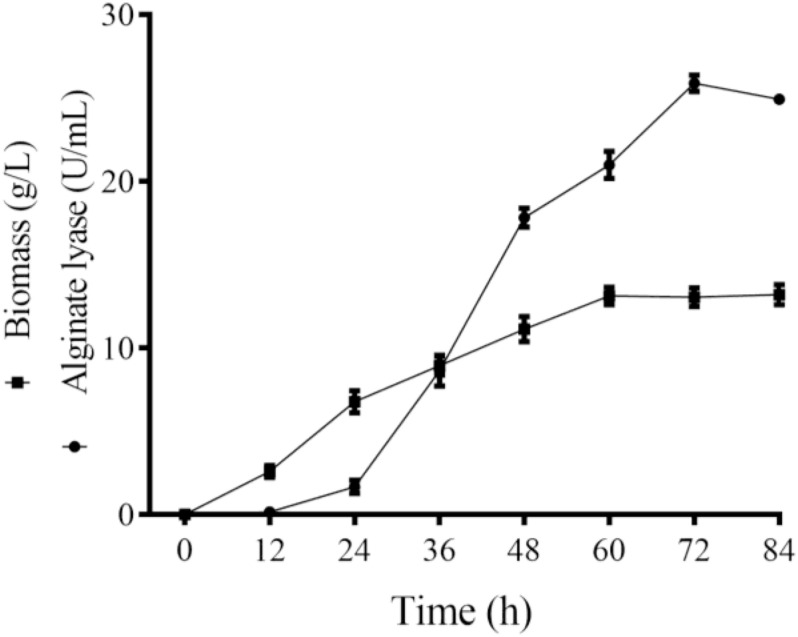
Time course of the Alyw203 activity secreted into the medium. Values are expressed as mean ± standard deviation (SD), *n* = 3.

**FIGURE 3:**
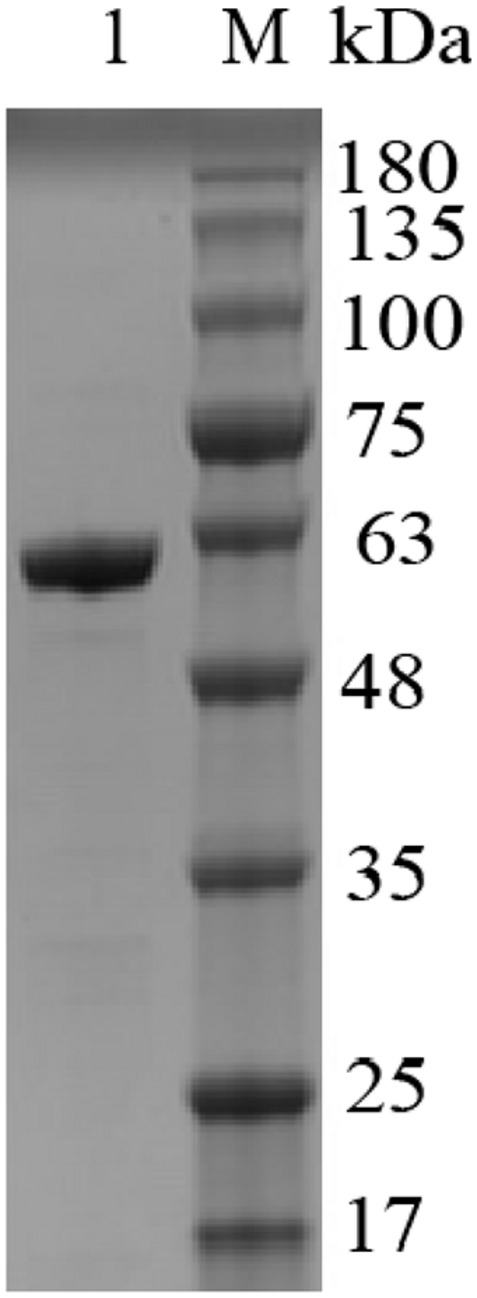
Analysis of Alyw203 by SDS-PAGE. Lane 1, purified Alyw203; Lane M, prestained protein ladder.

Majority of the reported alginate lyase expression is achieved in *Escherichia coli*. However, the extensive application of *E. coli* in industrial production is limited owing to the serious defects of poor secretion capability, presence of pyrogen, and endotoxin production (Miyamoto et al., 2009). In the current work, Alyw203 has been successfully expressed in *Y. lipolytica*, a heterologous host with excellent extracellular secretion ability (Madzak, 2015). Additionally, this system possesses the advantages of being safe, having high activity, and having no need to add antibiotics. As shown in Figure 2, after 72 h of culture in GPPB medium, the recombinant alginate lyase activity reached a peak of 25.9 U/ml, with a biomass of 12.5 g/L. Further SDS-PAGE analysis of purified Alyw203 protein shows that a clear band arises on the lane, meaning the Mw of Alyw203 is approximately 57.0 kDa (Figure 3), which is roughly in line with the theoretical Mw. Correspondingly, Zhuge et al. (2008) reported for the first time the cloning and functional expression of the *Bacillus subtilis* gene encoding alkaline pectate lyase in *Pichia pastoris*. The Mw calculated from the deduced amino acid sequence was similar to that of the protein secreted by yeast, approximately 43.6 kDa (Zhuge et al., 2008). Similarly, we previously reported that the alginate lyase gene Alyw201 from *Vibrio* was successfully expressed in the food-grade host *Y. lipolytica*. Studies have shown that the theoretical Mw of the enzyme and the measured actual Mw were 36.4 and 38.0 kDa, respectively, showing that there is little significant difference between them (Wang et al., 2020).

### pH Properties of Alyw203

**FIGURE 4:**
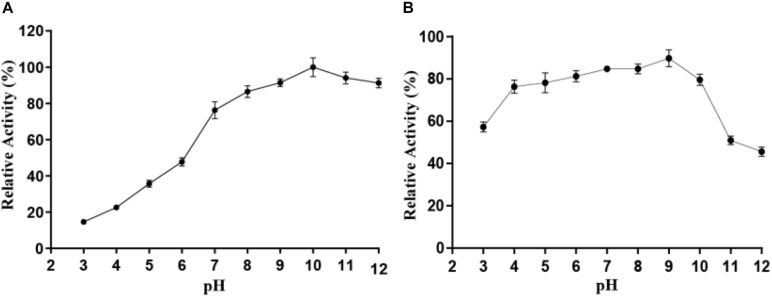
Effect of pH on the activity **(A)** and stability **(B)** of Alyw203. Values are expressed as mean ± standard deviation (SD), *n* = 3.

As displayed in Figure 4A, Alyw203 alginate lyase exhibits the highest catalytic activity at pH 10.0. As for the pH stability study of the present enzyme, it can be seen from Figure 4B that exceeding 40% of the activity is reserved after incubation in the pH range of 3.0–12.0.

### Temperature Properties and Heat Recovery of Alyw203

**FIGURE 5:**
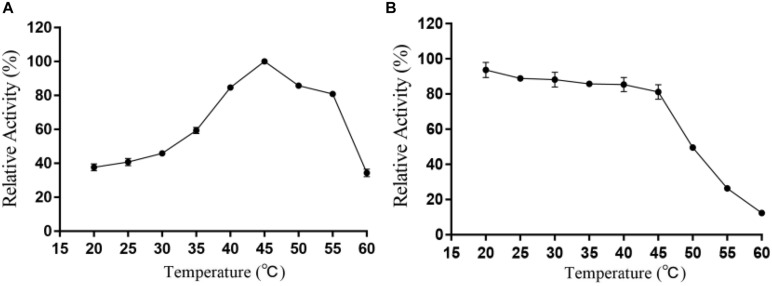
Effects of temperature on the activity **(A)** and stability **(B)** of Alyw203. Values are expressed as mean ± SD, *n* = 3.

It can be intuitively seen from Figure 5 that the highest activity of Alyw203 alginate lyase has been detected at 45°C, while it shows over 80% of the activity at 40–55°C. When the temperature exceeds 55°C, the corresponding enzyme activity drops sharply; meanwhile, the catalytic activity is lower than 60% of the highest activity at a temperature below 35°C. As for thermal stability, Alyw203 exhibits superior stability at temperatures below 45°C, retaining more than 80% of its highest activity in the subsequent incubation at 45°C for 3 h.

**FIGURE 6:**
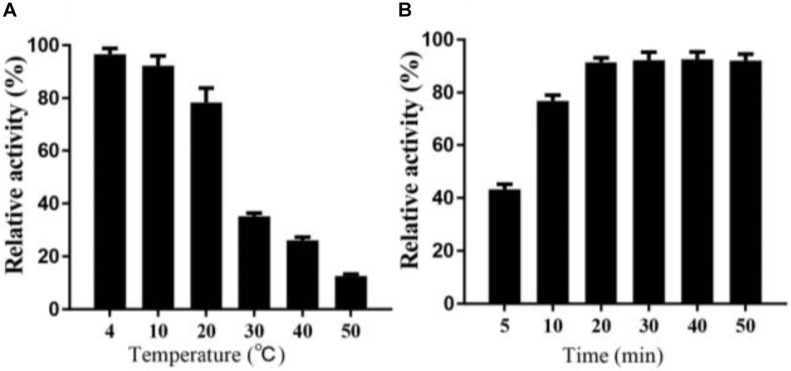
Heat recovery analysis of Alyw203. **(A)** Determination of the optimum incubation temperature for restoring activity of Alyw203 after 5 min of heat treatment. **(B)** Influence of incubation time on activity recovery of heat-treated Alyw203 at 10°C. Values are expressed as mean ± SD, *n* = 3.

Interestingly, in the process of determining the thermal stability of Alyw203, we find that the residual activity of the heat-treated enzyme after cold incubation can be significantly restored to a certain extent; that is to say, alginate lyase Alyw203 possesses a unique heat recovery performance. As shown in Figure 6A, the enzyme activity recovers to 96.3, 93.9, and 78.1% in the subsequent cultivation at 4, 10, and 20°C; while the incubation temperature exceeds 30°C, the activity recovery rate is less than 40%. For the purpose of further confirming the optimum incubation time for activity recovery, the current enzyme has been incubated at 10°C for different periods of time with the results illustrated in Figure 6B. As displayed in the figure, the activity of Alyw203 increases with the extension of the incubation time, which reaches the highest activity at 20 min and then basically remains unchanged. Interestingly, even after 5 and 10 min of incubation at 10°C, the activity of the current enzyme can be rapidly restored to the relative activity of 45.2 and 78.3%. These observations indicate that the current enzyme exhibits a thermal recovery character, which is conducive to the recovery of Alyw203 at low-temperature conditions.

### Effects of Ions and NaCl on Alyw203 Activity

**FIGURE 7:**
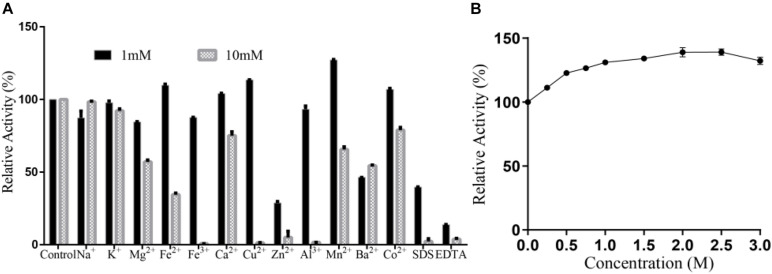
**(A)** Effects of ions on Alyw203. **(B)** Effects of NaCl on Alyw203. Values are expressed as mean ± SD, *n* = 3.

No significant activation effect of 1 mM metal ion on Alyw203 alginate lyase has been observed as displayed in Figure 7A, in which the relative activity increases to 138.0% merely under the condition of adding Mn^2+^. Nevertheless, 1 mM EDTA and SDS perform an obvious inhibitory effect on the present enzyme, wherein the relative activity is decreased to 14.7 and 62.8%, respectively. Under the condition of 10 mM, many ions including Fe^3+^, Cu^2+^, Zn^2+^, Al^3+^, SDS, and EDTA possess a powerful inactivation effect on the present enzyme, while no ions show an activation effect. Therefore, Alyw203 alginate lyase illustrates favorable ion tolerance. Additionally, as displayed in Figure 7B, the addition of NaCl (in the concentration range 0–3 M) slightly enhances the degradation activity of Alyw203 throughout the experiment, of which the highest activity achieved is 148.2% in the presence of 2 M NaCl.

### Product Analysis of Alyw203

**FIGURE 8:**
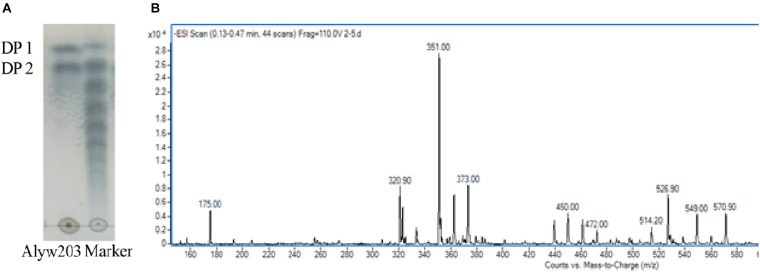
Degradation product analysis of Alyw203 by **(A)** TLC: Lane Alyw203, hydrolytic products of Alyw203; Lane Marker, standard alginate oligosaccharides; and **(B)** ESI-MS.

During the degradation process, the alginate polysaccharide is continuously degraded accompanied by the generation of oligosaccharides with various degrees of polymerization (DP). The degraded sample has been analyzed by TLC to determine the end product. It can be observed intuitively from Figure 8A that the two obvious spots of the final product on the TLC board are consistent with the alginate monosaccharide (DP1) and disaccharide (DP2) labels. ESI-MS (negative ion electrospray ionization mass spectrometry, Figure 8B) analysis further proves that these two spectra correspond to the Mws of unsaturated alginate monosaccharides (175.00 *m*/*z*) and disaccharides (351.00 *m*/*z*), indicating that the current Alyw203 enzyme effectively degrades alginate polymers into monosaccharides and disaccharides in an endo-type manner.

## Discussion

**FIGURE 9:**
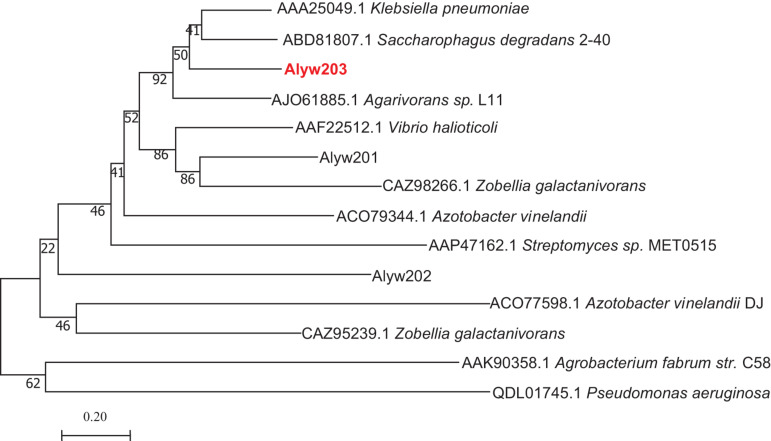
Phylogenetic relationship of the Alyw203 with other reported alginate lyases. Phylogenetic tree generated according to the ITS rDNA gene sequences with the neighbor-joining method. The number listed first represents the serial login number. The branching correlation numbers are bootstrapped values (confidence limits) that represent the replacement frequency of each amino acid residue.

In the detailed work of the present study, a novel alginate lyase, abbreviated as Alyw203, was successfully purified, cloned, and identified from *Vibrio* sp. W2. Sequence analysis displayed that the current enzyme was a novel alginate lyase pertaining to the PL2 family. As illustrated in Figure 9, Alyw203 was located on the same branch as the other two enzymes from *S. degradans* 2-40 (ABD81807.1) and *Agarivorans* sp. L11 (AJO61885.1). The results of multiple sequence comparison showed that Alyw203 contained three typical conserved regions: “QIH,” “RTELREMLR,” and “MYFKAG.” Moreover, since the “QVH” or “QIH” motifs played a pivotal part in the substrate selection of alginate lyase and the enzymes containing “QIH” motifs displayed a polyG preference (Huang et al., 2013; Swift et al., 2018), the current Alyw203 enzyme including the “QIH” conserved region revealed a predilection for polyG blocks. After comprehending the various characteristics of the alginate lyase shown in Figure 1, majority of the enzymes did display a predilection for polyG blocks (Wang et al., 2020). Interestingly, further sequence analysis indicated that the “QVH” motif may be an important indicator of polyM block preference, indicating that Alyw203 could be applied for the degradation of brown seaweed (Uchimura et al., 2010).

The current alginate lyase exhibited excellent pH stability. In general, majority of alginate lyases were inclined to catalyze degradation reaction within the small pH scope and neutral conditions as previously reported (Wang et al., 2019). Compared with the alginate lyase listed in Table 1, Alyw203 possessed catalytic activity within a wider pH range; that is, the current enzyme showed superior pH stability. For instance, ZH0-IV alginate lyase from *Sphingomonas* sp. (He et al., 2018) displayed the highest activity at pH 7.5, with a pH stability range of 6.0–9.0. A9m from *Vibrio* sp. A9m (Uchimura et al., 2010) possessed a neutral pH of 7.5 for optimum activity as well as a narrow pH stability range of 7.0–10.0. These enzymes had preferably neutral-pH conditions but had poor stability in alkaline environments. In addition, the Alyw203 and the reported pH-stable as well as highly active AlgNJ04 alginate lyase exhibited similar pH stability, which reserved over 80% of highest activity at an extensive pH from 4.0 to 10.0, indicating that the present enzyme could be applied for the extraction of active substances in brown algae (Zhu et al., 2018a).

**TABLE 1:** Comparison of Alyw203 performances with other alginate lyases.

Name	Source	Optimal pH/temperature (°C)	Temperature stable range (°C)	pH stable range	Product (DP)	References
NitAly	*Nitratiruptor* sp. SB155-2	6.0/70	40–80	4–7	3–5	Inoue et al., 2016
Alyw201	*Vibrio* sp.	8.0/35	4–10	4–10	2–6	Wang et al., 2020
TsAly6A	*Thalassomonas* sp.	8.0/35	0–30	6.6–8.95	2–3	Gao et al., 2018
TsAly7B	*Thalassomonas* sp.	8.0/20	0–25	7.3–8.6	2–3	Zhang et al., 2019
AlyPM	*Pseudoalteromonas* sp.	8.0/35	–	–	1	Chen et al., 2016
ZH0-IV	*Sphingomonas* sp.	7.5/35	25–42	6.0–9.0	1	He et al., 2018
AlyGC	*Glaciecola chathamensis*	7.0/30	–	–	1	Xu et al., 2017
Algb	*Vibrio* sp. W13	8.0/30	10–40	4.0–10.0	2–5	Zhu et al., 2015
A9m	*Vibrio* sp. A9mT	7.5/30	2–40	7.0–10.0	–	Uchimura et al., 2010
AlgNJU-03	*Vibrio* sp. NJU-03	7.0/30	10–40	6.0–9.0	2–5	Zhu et al., 2018b
Alyw203	*Vibrio* sp. W2	10.0/45	20–45	3.0–12.0	1–2	This study

The current enzyme possessed the highest activity detected at 45°C and showed a relative activity of more than 80% at 40–55°C. It is worth noting that Alyw203 alginate lyase displayed a unique heat recovery performance; that is, after cultivation for 20 min at 10°C, the activity of the enzyme boiled for 5 min could recover to over 90%. Considering that the inactivated enzyme could recover its activity after a short incubation period at 10°C, the thermal recovery properties of Alyw203 may effectively facilitate transportation and storage.

The study of the effect of ions on the present enzyme activity has shown that 1 mM metal ions could not effectively promote the activation of Alyw203, while Fe^3+^, Cu^2+^, Zn^2+^, and Al^3+^ exhibited obvious inactivation effects under the condition of 10 mM. Additionally, SDS and EDTA had remarkable inhibitory effects on the present enzyme. Alyw203 was not a strong NaCl-dependent enzyme. For many reported alginate lyases, a certain concentration of NaCl was a superior improver of enzyme activity. Like the activity of Aly08 from *Vibrio* sp., that of SY01 has been greatly increased by approximately eight times under the condition of 0.3 M NaCl. Similarly, activity of AlgM4 from *Vibrio weizhoudaoensis* has also been raised approximately seven times under the condition of 1 M NaCl. In comparison, the Alyw203 was not strongly dependent on NaCl. More interestingly, the activity of the current enzyme was still improved even in high concentrations of NaCl. In view of its superior salt tolerance, the Alyw203 alginate lyase was competent for specific production demands.

The main product of Alyw203 alginate lyase was DP1–DP2 oligosaccharide. Since alginate oligosaccharides are extensively used in the food, paper, and cosmetic industries (Falkeborg et al., 2014; Zhang et al., 2014; Qu et al., 2017), they have especially found important applications in the field of biomedical engineering in recent years; enzymatic production of single homogeneous oligosaccharides possessed a broad application prospect. However, majority of alginase degradation products found were a blend of DP2–DP6. For instance, the main products of Algb alginate lyase from *Vibrio* sp. W13 were DP2–DP5 (Zhu et al., 2015). Moreover, TsAly6A from *Thalassomonas* sp. (Gao et al., 2018) has DP2 and DP3 as its final degradation products. In comparison, the degradation products of the current alginate-degrading enzyme Alyw203 were concentrated in monosaccharide and disaccharide, which was conducive to further extraction as well as efficient production, showing potential drug application prospects.

## Data Availability Statement

The datasets presented in this study can be found in online repositories. The names of the repository/repositories and accession number(s) can be found in the article/supplementary material.

## Author Contributions

LL and HW: conceptualization, methodology, writing–original draft, and writing–review and editing. ZW: manuscript review and revision. ZZ and ZL: formal analysis and writing–original draft. LL: data curation and methodology. XJ and HC: funding acquisition and writing–review and editing. All authors have read and agreed to the published version of the manuscript.

## Conflict of Interest

The authors declare that the research was conducted in the absence of any commercial or financial relationships that could be construed as a potential conflict of interest. The handling editor declared a shared affiliation with one of the authors LL at the time of review.

## Publisher’s Note

All claims expressed in this article are solely those of the authors and do not necessarily represent those of their affiliated organizations, or those of the publisher, the editors and the reviewers. Any product that may be evaluated in this article, or claim that may be made by its manufacturer, is not guaranteed or endorsed by the publisher.

## Footnotes

1
http://www.cbs.dtu.dk/services/SignalP/

2
https://www.ncbi.nlm.nih.gov/cdd

3
http://web.expasy.org/compute_pi/
